# Two novel hierarchical homogeneous nanoarchitectures of TiO_2 _nanorods branched and P25-coated TiO_2 _nanotube arrays and their photocurrent performances

**DOI:** 10.1186/1556-276X-6-91

**Published:** 2011-01-18

**Authors:** Anzheng Hu, Cuixia Cheng, Xin Li, Jian Jiang, Ruimin Ding, Jianhui Zhu, Fei Wu, Jinping Liu, Xintang Huang

**Affiliations:** 1 Institute of Nanoscience and Nanotechnology, Central China Normal University, Wuhan 430079, P. R. China; 2 School of Physics and Electronic Engineering, Xiangfan University, Xiangfan 441053, Hubei, P. R. China

## Abstract

We report here for the first time the synthesis of two novel hierarchical homogeneous nanoarchitectures of TiO_2 _nanorods branched TiO_2 _nanotube arrays (BTs) and P25-coated TiO_2 _nanotube arrays (PCTs) using two-step method including electrochemical anodization and hydrothermal modification process. Then the photocurrent densities versus applied potentials of BTs, PCTs, and pure TiO_2 _nanotube arrays (TNTAs) were investigated as well. Interestingly, at -0.11 V and under the same illumination condition, the photocurrent densities of BTs and PCTs show more than 1.5 and 1 times higher than that of pure TNTAs, respectively, which can be mainly attributed to significant improvement of the light-absorbing and charge-harvesting efficiency resulting from both larger and rougher surface areas of BTs and PCTs. Furthermore, these dramatic improvements suggest that BTs and PCTs will achieve better photoelectric conversion efficiency and become the promising candidates for applications in DSSCs, sensors, and photocatalysis.

## Introduction

In current years, one-dimensional (1D) TiO_2 _nanostructure materials, especially nanotubular [1-3] and hierarchical [4-7] nanoarchitecture TiO_2 _nanotube arrays (TNTAs), have initiated increasing research interest owing to their intriguing architectures because they possess very high specific surface areas and a dual-channel for the benefit of the electrons transportation from interfaces to electrodes [7-13]. These nanostructure materials have shown very promising applications in dye-sensitized solar cells (DSSCs) [14-16], photocatalysis [17-19], photosplitting water [20,21], sensors [22,23], photoelectrochemical cells [24], and piezoelectronics [25]. However, as far as we are concerned, tremendous efforts have been conducted to improve the geometrical factors of the nanotube layers [8-13,26], to convert amorphous TiO_2 _nanotubes into different crystalline forms (i.e., anatase or rutile phase, or mixture phases of anatase and rutile) through high temperature annealing for high performance applications [27-29], and also many studies have devoted one's mind to change the crystal structure or chemistry composition of the tubes by modifying and doping [30-33]. There still remain many challenges to prepare and discuss the homogeneous modification of TNTAs, although the similar synthesis method of growing branched ZnO nanowires [34] and the decoration process of growing TiO_2 _nanoparticles on TiO_2 _nanotubes by a TiCl_4 _treatment [35] have been reported. Therefore, it is particularly valuable to seek some facile and high-efficiency method to synthesize the modification of TNTAs nanostructures for further specific surface area.

In this communication, we report for the first time the synthesis of two novel hierarchical homogeneous modification nanoarchitectures (i.e., P25-coated TNTAs, PCTs; and TiO_2 _nanorods branched TNTAs, BTs) via two-step method of electrochemical anodization and hydrothermal modification approach. The main precursors of modification are the P25 (Degussa, Germany) and titanium(IV) isopropoxide (TTIP of 95%). Erenow, the optimized nanoarchitecture TNTAs (with bigger pore diameter, longer length, and larger space among tubes) have been prepared by electrochemical anodization method. Interestingly, the as-synthesized BTs and PCTs with beautiful morphologies show both larger and rougher surface area, and these properties result in dramatic improvement of light-absorbing and charge-harvesting efficiency, which has been shown through the UV-Vis diffuse reflectance spectroscopic spectra and photoelectrochemical performances in this article.

### Experimental section

#### Fabrication of optimum nanoarchitecture TNTAs

In this article, TNTAs were prepared using a typical anodization approach [13]. Briefly, the fabrication process of the optimum nanoarchitecture TNTAs with bigger pore diameter, larger space among tubes and longer length was described as follows, Titanium foil samples, about 200 μm × 2 cm × 3.5 cm (Purity≥99.6%, from ShengXin non-ferrous metal Co., LTD, Baoji, Shanxi, China) were cleaned with soap, acetone, and iso-propanol before anodization. A two-electrode configuration was used for anodization, with Ti foil as the anode, and platinum foil as the cathode. A 99.7% pure Ti foil (0.2 mm thickness, 2 × 3 cm^2^) was immersed in the electrolyte containing 0.35 wt% NH_4_F (85% Lactic Acid) and 10 vol.% DMSO (dimethyl sulphoxide: purity ≥99.0%) at a 45 V constant potential for 9 h. Thus we obtained the amorphous TNTAs, and then the as-prepared TNTAs were annealed at 400°C for 1.5 h for further use.

#### Synthesis of hierarchical homogeneous nanoarchitecture BTs

The BTs were obtained via a modification process of growing TiO_2 _nanorods on the as-prepared TNTAs by conventional hydrothermal growth method. Briefly, the as-prepared TNTAs were immersed in a beaker with growth solution, this solution was consisted of 90 mL of 0.8 M HCl (36-38%) with constant stirring at 25°C for about 15 min. After that, 6 mL of TTIP of 95% as precursor was dropped (0.16 μL/s) in mixture solution, kept stirring for 1 h [7,32,33], and then the beaker was sealed and heated at 95°C for 9 h, with slight stirring maintained for the entire heating process to grow TiO_2 _nanorods on the TNTAs. After the reaction, the reactant was cooled freely to room temperature and washed several times with ethanol and distilled water, and the as-prepared BTs were obtained. The BTs were finally achieved through annealing in a muffle furnace at 400°C for 2 h.

#### Fabrication of hierarchical homogeneous nanoarchitecture PCTs

We fabricated PCTs via a hydrothermal approach of coating P25 on the as-prepared TNTAs. About 0.4 g P25 (Degussa, Germany) was put into a beaker with 300 mL of distilled water, then they were mixed through vigorous magnetic stirring and ultrasonicating alternately at room temperature more than 5 times (about 10 min per time), After that, the mixed solution was kept state static more than 3 h, and then transferred into a Teflon-lined autoclave (80 mL), in which the as-prepared TNTAs were suspended. The autoclave was sealed and heated at 80-120°C for 12 h to coat P25 on the TNTAs, and then it was cooled freely to room temperature and washed several times with distilled water, thus the as-prepared PCTs were obtained. Finally, the PCTs were fabricated after the as-prepared PCTs were annealed at 400°C for 2 h.

### Characterization

The crystal structures of the as-synthesized samples were firstly determined by using a Bruker D8 advance X-ray diffractometer (XRD, Cu Kα radiation; λ = 1.5418 Å). Then the morphologies were observed by field-emission scanning electron microscopy (FESEM, JOEL, JSM-6700F), and transmission electron microscopy (TEM and HRTEM, JEM-2010FEF; 200 kV). Photoelectrochemical experiments were carried out using a three-electrode configuration (CH instruments, CHI 660C) with a Pt wire counter electrode, a reference saturated calomel electrode and a working electrode. The all samples used as working electrodes were illuminated with a 150~350 W adjustable xenon lamp (from Shanghai Lansheng Electronics Co., LTD., Model, XQ350W). The measured light irradiance was approximately 100 mW/cm^2^, and the scan rate was 100 mV/s

## Results and discussion

**Figure 1:**
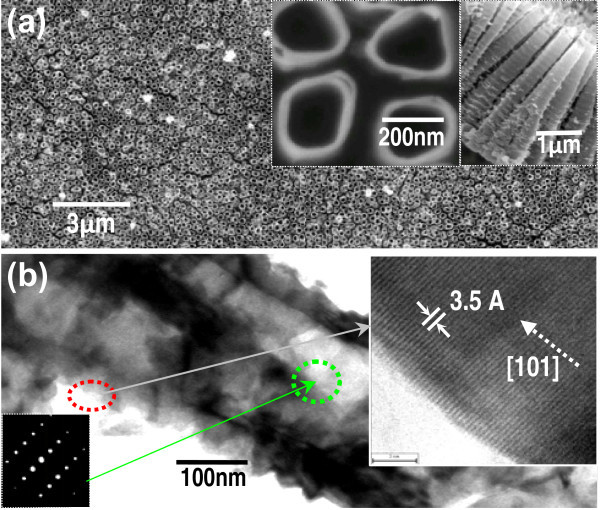
**Characterization images of the TNTAs see (a) and (b): (a) **Low-magnification FESEM, insets are enlarged FESEM images of the top view and cross-section of its typical tubes, respectively; **(b)**. TEM image of the individual TiO_2 _nanotube, insets are its HRTEM and SAED images of the marked areas, respectively.

In this study, the two-step method is used to synthesize the BTs and PCTs. The first step is the fabrication of the optimize nanoarchitecture TNTAs [36,37]. From Figure 1, it can be found that the TNTAs show very nice highly ordered, self-organized, and free-standing morphologies, and the optimize geometrical architectures (average external diameter, 350 nm; tube length, 3.5 μm; wall thickness, 10 nm; and space among tubes, 60 nm), and also show at least local single-crystalline status. These characterizations can be observed from the FESEM images of the top view and cross-section of the TNTAs shown in Figure 1a and the TEM, SAED, and HRTEM images in Figure 1b.

**Figure 2:**
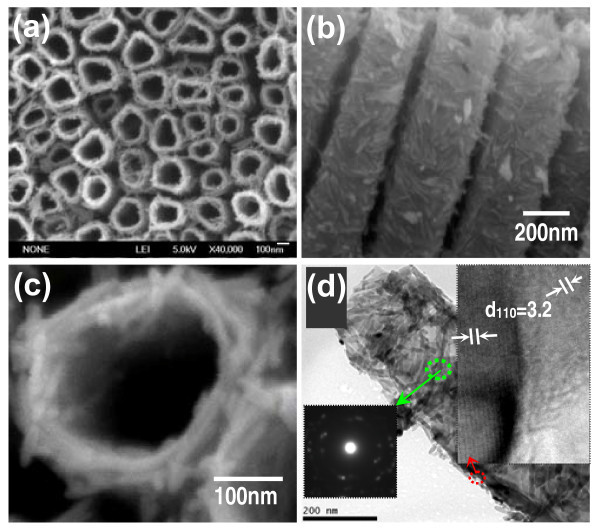
**FESEM images of (a) top view, (b) cross-section view, (c) high-magnification top view of BTs**. **(d) **TEM image of a typical individual branched TiO_2 _nanotube shown in (a); insets are its SAED and HRTEM images of the marked areas, respectively.

**Figure 3:**
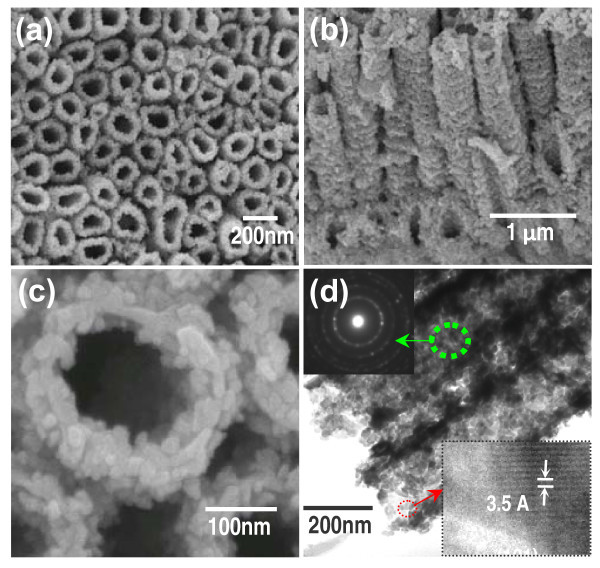
**FESEM images of (a) top view, (b) cross-section view, (c) high-magnification top view of PCTs**. **(d) **TEM image of several typical PCTs shown in (a); insets are their SAED and HRTEM images of the marked areas, respectively.

**Figure 4:**
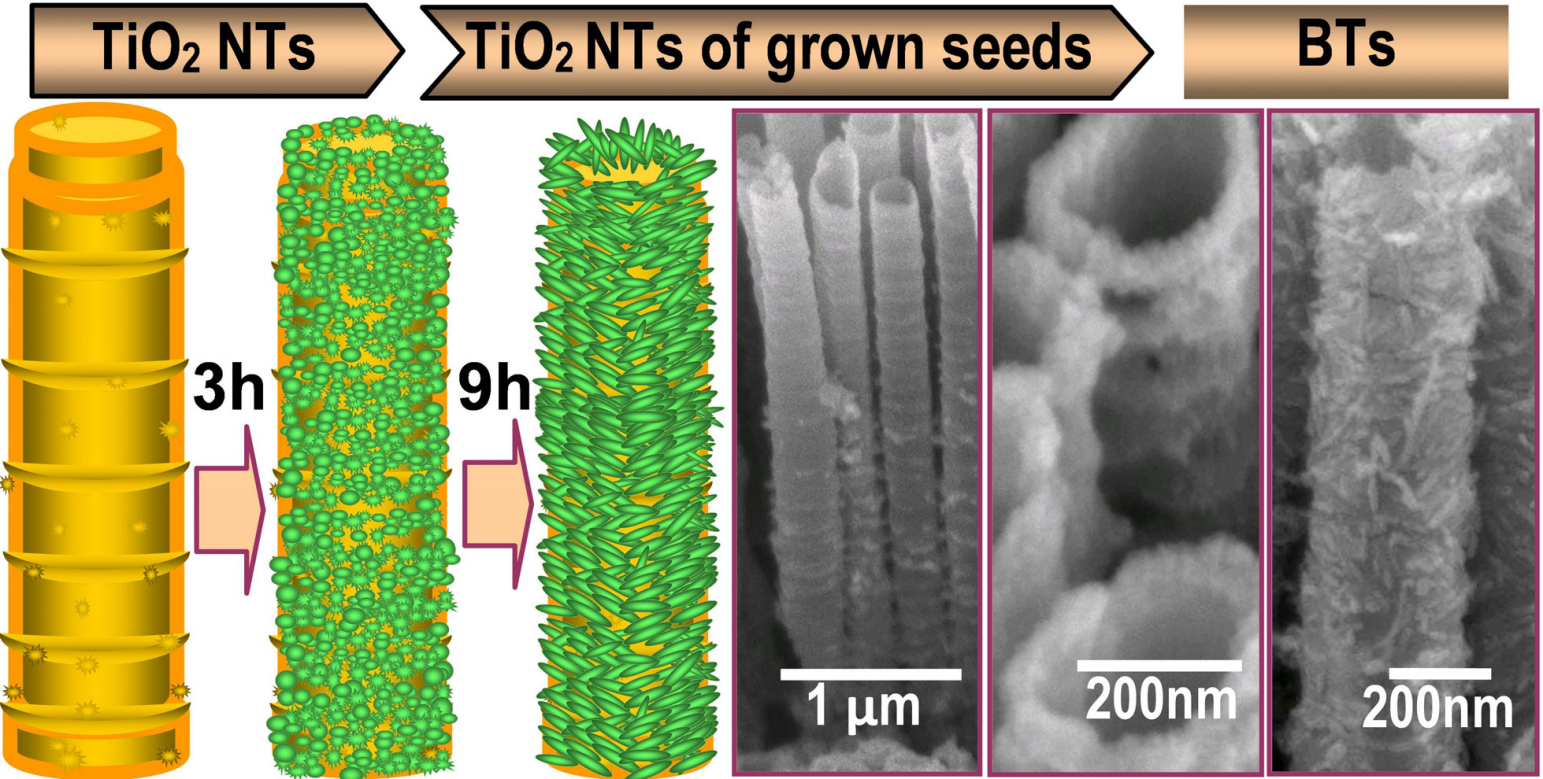
**The section on the left is the morphology evolution of BTs, and their corresponding FESEM images are on the right**.

The second step is the synthesis of BTs and PCTs using hydrothermal modification method. In brief, they were obtained from growing branched TiO_2 _nanorods and coating P25 on the pre-prepared TNTAs via hydrothermal modification process, the images of obtained BTs and PCTs are shown in Figures 2 and 3, respectively. As for the BTs, the mechanism of the formation of TiO_2 _crystal nucleus and growth of the anisotropic 1D nanocrystalline TiO_2 _nanorods, and their corresponding FESEM images are depicted in Figure 4. From schematic diagram of the morphologies evolution of the BTs and the FESEM images, it is clearly observed that more and more TiO_2 _nanocrystal nucleus were firstly formed on the rough surfaces of the TiO_2 _tubes with special bamboo structures, many rings and attached particles, these special structures and morphologies are the probable cause of crystal nucleus formed. And then the nucleus gradually grew up and became increasing TiO_2 _nanorods along the backbones of the TiO_2 _tubes, along with a small quantity of free-grown rods random adhered to the backbones of the tubes. Thus these TiO_2 _nanorods made BTs have both larger and rougher surface area [7,34]. Furthermore, the same conclusion can also be confirmed by the top view FESEM images showed in Figure 2a, c, the cross-sectional view in Figure 2b, the TEM image of a individual branched TiO_2 _nanotube in Figure 2d. And the insets in Figure 2d are the SAED pattern and the HRTEM images, which show the BTs are evident polycrystalline.

Figure 3 is the characterization of another homogeneity nanostructure (the PCTs). Figure 3a is the top view FESEM image of the PCTs. A cross-sectional view in Figure 3b shows that the length of the tubes is the same as that of TNTAs (about 3.5 μm) and the P25 nanoparticles are densely grown on the whole surface (including inside and outside) of the TiO_2 _tubes. And the top view of the PCTs with many attached P25 particles is clearly shown by the high-magnification FESEM image in Figure 3c. Meanwhile, Figure 3d shows the PCTs' TEM image, and its inset of the HRTEM image shows the (101) crystal facet and the 0.35 nm interplane distance of a typical anatase TiO_2 _while the another inset of the SAED pattern shows that the PCTs are polycrystalline structure [24]. The growth mechanism of the PCTs is mainly dependent on the special structures and morphology of TNTAs, especially its bigger pore diameter, larger space among tubes, and rough surface. Moreover, annealing plays an important role in the process of transforming the P25 on the TiO_2 _tube surface from attached state into crystallization state.

**Figure 5:**
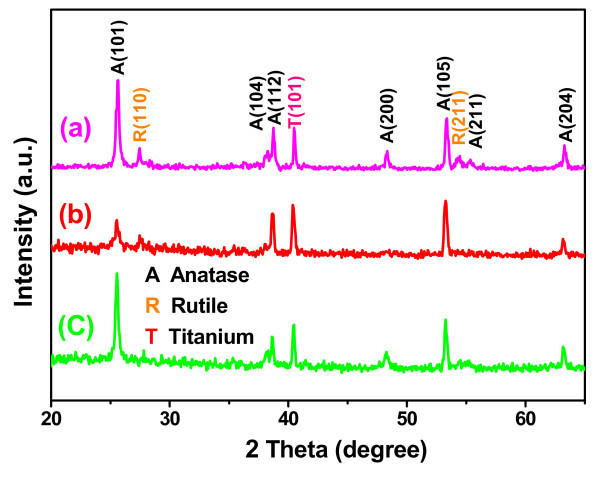
**XRD patterns of (a) BTs, (b) PCTs, and (c) TNTAs**.

Otherwise, the X-ray diffraction (XRD) patterns in Figure 5a, b, c are also employed to characterize the properties of the obtained samples. We can find that the diffraction peaks of the samples (b, c) and the dominant diffraction peaks of the samples (a) match well with the crystal structure of the anatase TiO_2 _phase (JCPDS 21-1272) [38] except for one peak of the Ti (101). The main reason can be attributed to thermal treatment temperature of no more than 400°C for 2 h. It is noteworthy that the two peaks [R (110) and R (211)] in Figure 5a just match with the crystal structure of the rutile TiO_2 _nanorod (JCPDS no. 21-1276) [7,12], this comes from those rutile TiO2 nanorods grown on the TNTAs.

On the basis of the above observations and structural analyses, we conclude that both of the BTs and PCTs can provide larger and rougher surface areas than the TNTAs compared with the arrays of same geometrical size and quantity [7,34,35]. As a result, this larger and rougher surface areas are favorable to improve light-absorbing and charge-harvesting efficiency and to absorb more dye for better photoelectric conversion efficiency and better applications such as photocatalysis, sensors, etc. Moreover, it is also found that the growth length and density of the TiO_2 _nanorods of the BTs can be readily controlled by adjusting the growth time and the concentration of growth solution, and that the density of the coated P25 particles can also be controlled through changing the coating time and the concentration of coating solution.

**Figure 6:**
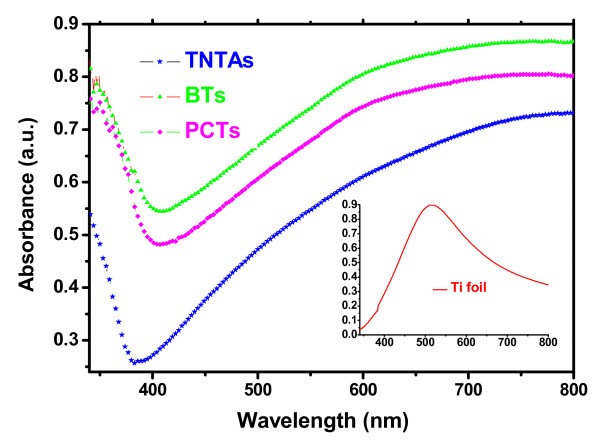
**UV-Vis diffuse reflectance spectra of the samples (TNTAs, PCTs, BTs, and inset, Ti foil)**.

Figure 6 shows the UV-Vis diffuse reflectance spectra of three samples (TNTAs, PCTs, and BTs) and Ti foil. Comparing to the UV-Vis absorption spectrum of the TNTAs, the absorption edges of the samples (PCTs and BTs) displayed appreciable shifts (BTs is a little bit larger than PCTs) to visible region revealing some decreases in their band gaps. This conclusion is mainly consistent with the above discussions and the previous studies [39-41]. Simultaneously, it can also be found that the absorption intensity of each sample (TNTAs, PCTs, and BTs) is gradually increasing after their absorption peaks. The cause for this effect mainly comes from absorption effect of the annealed (400°C, 2 h) Ti foil substrate to visible (see the inset in Figure 6). Otherwise, the general UV-Vis absorption spectra only reflect the intrinsic optical property for the bulk of a solid. However, the actual absorption spectrum of a photocatalyst is an overlapping result of intrinsic and extrinsic absorption bands [42].

**Figure 7:**
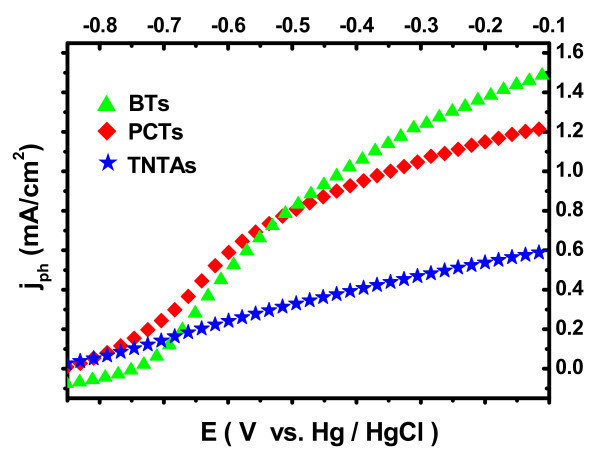
**Variation curves of photocurrent densities versus measured potentials for three different photoanodes (TNTAs, PCTs, and BTs) in 1 M KOH electrolyte**.

Furthermore, Figure 7 clearly shows the comparison curves of the photocurrent densities versus applied potentials for three different TiO_2 _photoanodes (TNTAs, BTs, and PCTs) under Xe lamp irradiation (100 mW/cm^2^) in 1 M KOH electrolyte [43]. It can be observed that the values of the photocurrent densities of BTs and PCTs are dramatically greater than that of TNTAs. At -0.11 V and under the same illumination conditions, the photocurrent density of BTs shows more than 1.5 times higher than that of TNTAs while PCTs versus TNTAs is more than 1 times higher. These experimental results are well consistent with the effect from above UV-Vis diffuse reflectance spectra. They suggest that the BTs and PCTs used as photoanodes can harvest more solar light and more photogenerated charge than that of the TNTAs with the same geometrical structure. In addition, the photocurrent densities of the BTs and PCTs also show a steeper increase when their applied potentials are over -0.7 V. Thus as for the BTs and PCTs, e^-^-h^+ ^pairs induced by photon absorption are split more readily compared with the TNTAs. The conclusion mainly results from the fact that more incident photons are absorbed on the electrode with larger and rougher space area [44].

## Conclusion

In summary, we have reported here the fabrication of two novel hierarchical homogeneous nanoarchitectures of BTs and PCTs with larger and rougher surface areas via facile hydrothermal modification process. Based on the investigation of the photocurrent densities versus applied potential, the photocurrent density of BTs, at -0.11 V and under the same illumination conditions, shows more than 1.5 times higher than that of TNTAs while PCTs versus TNTAs is more than 1 times higher. On the basis of the results and discussion, we conclude that the dramatically improved photocurrent densities of the BTs and PCTs used as photoanodes are mainly due to their better incident photons and photogenerated charge-harvesting capability compared to TNTAs resulting from their further enhanced and rough surface areas. As a result, our study will also provide a new approach in conformating hierarchical homogeneity nanostructure materials and presenting two kinds of promising candidates for applications in DSSCs, sensors, and photocatalysis.

## Abbreviations

BTs: branched TiO_2 _nanotube arrays; DSSCs: dye-sensitized solar cells; FESEM: field-emission scanning electron microscopy; PCTs: P25-coated TiO_2 _nanotube arrays; TEM: transmission electron microscopy; TNTAs: TiO_2 _nanotube arrays; XRD: X-ray diffractometer.

## Competing interests

The authors declare that they have no competing interests.

## Authors' contributions

AH presided over and fully participated in all of the work. CC and XL participated in the preparation of the samples. JJ and RM participated in the revision of the manuscript and the statistical analysis of experimental data. JH and FW participated in the investigation of the photocurrent performances. XT and JP participated in the design and idea of the study. All authors read and approved the final manuscript.
